# Role of Jasmonic Acid Pathway in Tomato Plant-*Pseudomonas syringae* Interaction

**DOI:** 10.3390/plants9020136

**Published:** 2020-01-22

**Authors:** Loredana Scalschi, Eugenio Llorens, Pilar García-Agustín, Begonya Vicedo

**Affiliations:** Grupo de Bioquímica y Biotecnología, Departamento de Ciencias Agrarias y del Medio Natural, Universitat Jaume I de Castellón, Avenida de Vicent Sos Baynat, s/n, 12071 Castellón de la Plana, Spain; ellorens@uji.es (E.L.); garciap@uji.es (P.G.-A.); bvicedo@uji.es (B.V.)

**Keywords:** Jasmonic acid, *OPR3*, *Pseudomonas syringae*, OPDA

## Abstract

The jasmonic acid pathway has been considered as the backbone of the response against necrotrophic pathogens. However, a hemi-biotrophic pathogen, such as *Pseudomonas syringae*, has taken advantage of the crosstalk between the different plant hormones in order to manipulate the responses for its own interest. Despite that, the way in which *Pseudomonas syringae* releases coronatine to activate jasmonic acid-derived responses and block the activation of salicylic acid-mediated responses is widely known. However, the implication of the jasmonic intermediates in the plant-*Pseudomonas* interaction is not studied yet. In this work, we analyzed the response of both, plant and bacteria using *SiOPR3* tomato plants. Interestingly, *SiOPR3* plants are more resistant to infection with *Pseudomonas*. The gene expression of bacteria showed that, in *SiOPR3* plants, the activation of pathogenicity is repressed in comparison to wild type plants, suggesting that the jasmonic acid pathway might play a role in the pathogenicity of the bacteria. Moreover, treatments with JA restore the susceptibility as well as activate the expression of bacterial pathogenicity genes. The observed results suggest that a complete jasmonic acid pathway is necessary for the susceptibility of tomato plants to *Pseudomonas syringae*.

## 1. Introduction

A network of plant hormones that trigger specific defenses, orchestrate plant innate resistance against pests and pathogens. Oxylipins are considered to be crucial compounds for plants, since they play important developmental roles and are also involved in plant defense mechanisms. This family of molecules includes a vast array of bioactive metabolites that are generated from membrane lipids as a result of lipid peroxidation [1,2]. Among them, jasmonates are a family of signaling molecules that act as phytohormones and regulate different processes that are related to plant development, such as tuber and trichome formation, leaf senescence, or reproductive development. Moreover, these phytohormones also regulate symbiotic interactions and plant responses to wounding, and defenses against insects and pathogens [3,4].

Jasmonic acid (JA) has been considered for a long time as the end product of the pathway and, therefore, considered as the bioactive hormone. However, subsequently, the involvement of other molecules, such as the isoleucine (Ile) conjugate of JA, jasmonoyl isoleucine (JA-Ile), has been demonstrated. This molecule possesses a strong biological activity being, perhaps, the best-characterized family member. The conjugation of JA to Ile takes place in the cytoplasm and it is catalyzed by the amino acid conjugate synthetase JASMONATE RESISTANT1 (JAR1) [5]. JA-Ile can bind to the JA-receptor, COI1. The interaction JA-Ile/COI mediates the ubiquitin-dependent degradation of JAZ repressors, which results in the activation of JA-dependent gene expression controlled by MYCs and ethylene response factor (ERF) [6,7].

Besides the above-mentioned molecules, the precursor of JA, cis-(+)-12-oxo-phytodienoic acid (OPDA), has also been identified as a signaling molecule controlling a set of genes independent of JA/JA-Ile that includes COI1-dependent as well as COI1-independent genes [8,9,10,11]. It has been demonstrated that OPDA is involved in a multitude of plant developmental processes like seed dormancy, germination, and embryogenesis [12]. However, it also plays a defensive role in plants, since OPDA biosynthesis is induced upon abiotic and biotic stress. Analysis using mutants that are deficient in the biosynthesis of JAs revealed that OPDA functions as a signaling molecule in the wounding response [9]. Moreover, OPDA confers resistance of Arabidopsis plants against different pathogens in the absence of JA/JA-Ile [8,13], while rice mutants deficient in OPDA showed altered defense against the blast fungus *Magnaporthe oryzae* [14]. Recent studies have also suggested that OPDA is involved in enhancing callose accumulation in host plants to limit pathogen infection [15,16].

Although the mechanisms that underlie COI1 independent OPDA signaling are still not elucidated, it is known that the presence of an α,β -unsaturated carbonyl group, as in OPDA, is able to induce the expression of stress-related genes that belong to the glutathione-S-transferase family. This set of genes is independent of those that would be activated by JA, which suggests a COI1-independent signaling cascade that would not require a specific OPDA receptor [2,9]. Furthermore, it was demonstrated that the interaction of OPDA with the plastidic Cyclophilin20-3 (CYP20-3) contributes to OPDA signaling after wounding [17].

Maynard et al. recently reviewed current knowledge regarding OPDA crosstalk with other phytohormones [18]. Overall, the authors conclude that the regulation of the oxylipin pathways by salicylic acid (SA) is controversial and it differs among plant species. On the other hand, the cross-talk between OPDA and abscisic acid (ABA) is clearer, since both hormones are involved in similar processes, such as defense against pathogens, induction of stomatal closure, and redox homeostasis [8,9,19].

*Pseudomonas syringae* pv *tomato* (*Pst*) is a hemibiotroph bacterium that infects a wide variety of plants, being able to live both on the surface and in the apoplast of the plant. The bacteria must overcome the plant’s innate immune response in order to develop colonies in the apoplast and infect neighboring tissues. To achieve this, *P. syringae* produces the toxin coronatine (COR) that alters plant responses and induces chlorosis on a wide variety of plant species. COR consists of two different structural components that function as biosynthetic intermediates: the polyketide coronafacic acid (CFA) and coronamic acid (CMA). CMA derives from L-alloisoleucine, whereas CFA is synthesized from cyclopentenone, which is modified by the genes of the CFA operon. Finally, coronafacate ligase joins CFA and CMA with an amide linkage to form COR [20]. Once the molecule is released by the bacteria, it moves into the plant cell through diffusion. This toxin binds to COI receptors activating JA derived responses in the same way that the JA-Ile molecule does due to similar structure between JA-Ile and COR. Additionally, COR plays a role in opening stomata, helping, this way, the bacteria to enter the leaf [21].

Besides COR synthesis, the ability of *Pst* to grow and cause diseases in plants is also dependent on the injection of multiple effector proteins into plant cells via the type III secretion system (T3SS) [22], The T3SS apparatus consists of a basal body which spans both bacterial membranes and an extracellular needle or pilus. The main component of the pilus is the HrpA protein. The T3SS is encoded by the hrp (HR and pathogenicity) and hrc (HR and conserved) genes, which are contained in the hrp/hrc cluster. The expression of most T3SS genes is activated by the alternative sigma factor HrpL. T3SS is used by the bacteria to inject type III effectors into host plant cells to suppress both pathogen-associated molecular pattern (PAMP)-triggered immunity (PTI) and effector-triggered immunity (ETI). *Pst* produces around 35 effectors with various enzymatic activities, like cysteine proteases (AvrPphB and AvrRpt2), mono-ADP-ribosyltransferases (HopU1 and HopF2), phosphothreonine lyase (HopAI1), E3 ligase (AvrPtoB), etc. [23,24].

We have recently demonstrated that *OPR3*-silenced tomato plants (*SiOPR3*) are more susceptible to the necrotrophic pathogen *Bortytis cinerea* when compared with the WT plants [16]. The characterization of the *SiOPR3* plants revealed a role of OPDA in the resistance of tomato plants to *B. cinerea* that is independent of JA-Ile and pointed to a direct relationship between the OPDA levels and callose deposition.

However, the role of this molecule in the pathosystem tomato-*Pseudomonas* has not been determined yet. The goal of this work was to study the behavior of *SiOPR3* upon infection with *Pst*, a hemibiotrophic bacteria to gain a better understanding of the potential role of OPDA in plant defense and, therefore, a pathogen with a lifestyle and virulence mechanisms other than the necrotrophic *Botrytis cinerea*. The results show that *SiOPR3* are more resistant to this pathogen when compared with WT plants. Furthermore, the resistance of *SiOPR3* tomato plants is accompanied by an increase in OPDA and SA levels and a decrease in the virulence of the bacteria, as demonstrated by the bacterial gene expression analysis.

## 2. Results

### 2.1. SiOPR3 Plants Are More Resistant to Pseudomonas syringae

**Figure 1:**
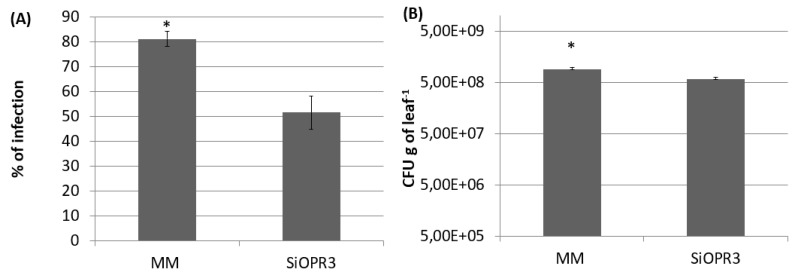
SiOPR3 transgenic plants have increased resistance to *Pseudomonas syringae*. Four-week-old tomato plants cv. Moneymaker (MM) and SiOPR3 were inoculated with *Pst*, as described in Materials and Methods. Seventy-two hours after inoculation, the disease rate was evaluated by visual observation to determine the percentage of dark brown spots on the leaf surface (**A**) and by recounting the bacterial populations through plating in agar-King’s B medium (**B**). The data show the average values ± standard error (*n* = 20). The asterisks represent statistically significant differences (*p* < 0.05; least-significant difference test).

The plants with silenced *OPR3* (*SiOPR3* tomato plants) were inoculated with *Pst* to determine the role of OPDA in the resistance against biotrophic pathogens. Silenced plants developed fewer symptoms of disease at 72 hpi, since an average reduction of approximately 25% in disease symptoms was observed in these plants in comparison with the damage that was observed in wild type plants (Figure 1A). In the same way, *SiOPR3* tomato plants showed statistically significant reductions in the size of the bacterial population when compared to those that were observed in WT plants. The study of the CFU showed levels of bacterial population of around 7.44 × 10^9^ CFUx g^−1^ in wild type plants and of 6.8 × 10^8^ CFUx g^−1^ of leaf for *SiOPR3* plants (Figure 1B). Both of the results confirm that *SiOPR3* plants are more resistant against *Pst,* which suggests that *OPR3* plays an essential role in tomato defense against biotroph attack.

### 2.2. SiOPR3 Plants Accumulate More OPDA Upon Infection

**Figure 2:**
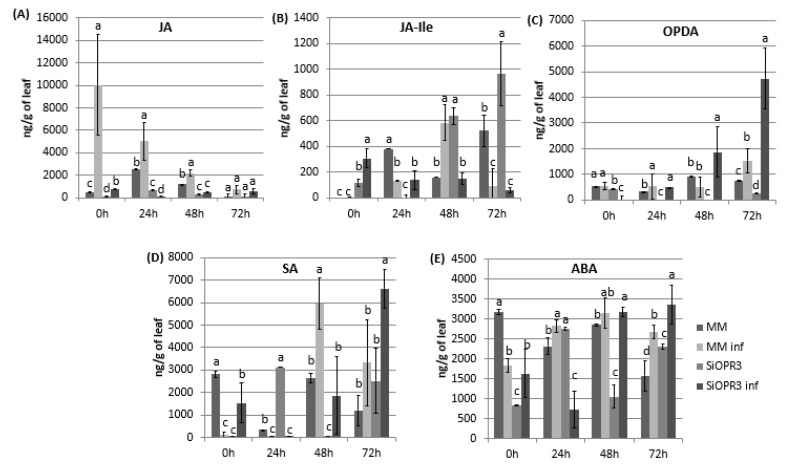
Hormone levels in MM and SiOPR3 plants upon *Pst* infection. Leaves were collected at 0, 24, 48 and 72 hpi and JA (**A**), JA-Ile (**B**), cis-(+)-12-oxo-phytodienoic acid (OPDA) (**C**), salicylic acid (SA) (**D**), and abscisic acid (ABA) (**E**) levels were determined by ultra-performance liquid chromatography (UPLC)-mass spectrometry. Data show the average of three independent experiments of a pool of 10 plants per experiment ± SE. Different letters indicate statistically significant differences between treatments at the same time point (*p* < 0.05; least-significant difference test).

The hormonal levels were analyzed in WT and transgenic tomato lines both in the absence or presence of the infection in order to check the implication of silencing in the hormonal response of the plant against infection with *Pst*. Regarding JA pathway, we measured the levels of JA, JA-Ile, and OPDA. The results showed that the levels of JA were similar between wild type and *SiOPR3* plants in the absence of inoculation (Figure 2A). However, in the presence of the bacteria, a strong increase in the levels of JA in WT plants at 0 h was observed and it gradually reduces during the experiment. On the other hand, the *SiOPR3* plants showed no changes in the level of JA after inoculation during the experiment, which corroborates the silencing of the pathway (Figure 2A).

The levels of JA-Ile, which is considered to be the bioactive molecule of jasmonic acid pathway, showed a completely different pattern. In the absence of challenge inoculation, silenced plants showed an increasing level of JA-Ile during the experiment (Figure 2B). However, wild type plants accumulate JA-Ile at 48 h after inoculation. Moreover, in both cases, the levels of JA-IIe in the inoculated plants are reduced at 72 h when compared with non-inoculated plants (Figure 2B).

Interestingly, the accumulation of OPDA was only observed in silenced plants after inoculation. This accumulation increases over time reaching the highest values at 72 h post-inoculation. In wild type plants, a small increase of OPDA was only observed at 72 h post-inoculation, whereas no differences were observed for the rest of the studied time points (Figure 2C).

It is known that salicylic acid pathway mediates the resistance against biotrophic pathogens, such as *Pst*. Therefore, SA levels were determined in the same samples in which JA, JA-Ile, and OPDA were measured (Figure 2D). After infection, the WT plants showed significantly increased SA levels at 48 h that are slightly lower at 72 h. Moreover, surprisingly, in *SiOPR3*, the accumulation of SA is only enhanced at 72 hpi (Figure 2D).

Cross talk between OPDA and ABA has been deeply studied and it is suggested that both of the hormones are involved in similar processes [18]. Therefore, we next analyzed the levels of abscisic acid and observed that, at short time points, the ABA levels in uninfected WT plants were higher than those observed in the other plants (Figure 2E). Moreover, although the levels of ABA in the silenced and inoculated plants considerably decrease at 24 hpi, they went back up at 48 hpi and were maintained over time (Figure 2A). Interestingly, at 72 hpi, the increase in ABA levels was more pronounced in *SiOPR3* infected plants than in WT infected plants and seems to correlate with the increase that was observed in OPDA levels at this time point.

Moreover, *Pst* was incubated in LB medium in the absence or presence of different concentrations of OPDA (0.1, 1, and 5 µM) being the lowest concentration equal to the highest concentration measured in planta in order to check whether the OPDA present in the plant could act directly to impair the bacterial growth. The addition of OPDA did not reduce the growth of the bacteria when compared with the control at none of the tested concentrations. Therefore, a direct effect of OPDA on the bacteria was ruled out (data not shown).

### 2.3. Marker Genes for JA, SA and ABA Signaling Pathways Are More Strongly Induced in SiOPR3 Plants than in Wild Type Plants upon Infection

The results that were obtained for hormonal analysis prompted us to analyze the expression pattern of the marker genes for JA synthesis pathway (*AOC, OPR3*) and for SA (*PR1, PR5*) and ABA (*ASR1*) signaling pathways in the leaf samples of WT and *SiOPR3* tomato plants. Gene expression was determined at this time point since major changes in hormone levels were observed at 72 h.

**Figure 3:**
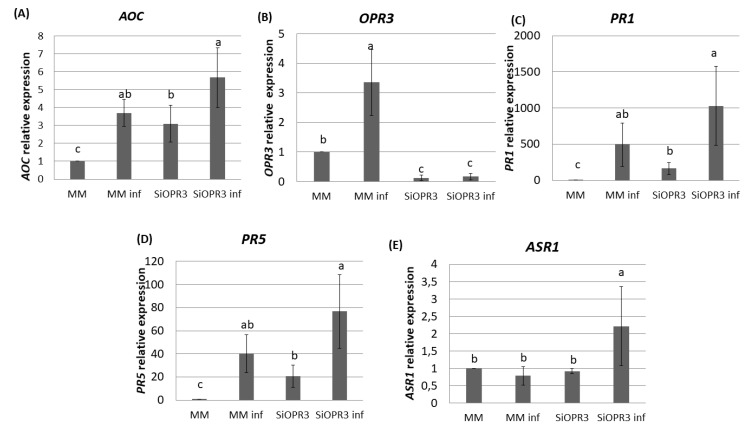
Gene expression profile of plant defense pathways in MM and SiOPR3 plants upon *Pst* infection. MM and SiOPR3 plants were grown and inoculated as described in Materials and Methods. The expression of the *AOC* (**A**), *OPR3* (**B**), *PR1* (**C**), *PR5* (**D**), and *ASR1* (**E**) genes were analyzed in cDNA from MM and SiOPR3 plants at 72  h post-inoculation. The results were normalized to the *EF1α* gene expression measured in the same samples. The results show the average values of three independent experiments with similar results ± standard error (*n* = 3).

The silenced plants showed an expression of the *AOC* gene 35% higher after challenge inoculation as compared with wild type plants (Figure 3A). These results are according to the higher accumulation of OPDA that was observed in silenced plants after challenge inoculation. Moreover, in the absence of inoculation, *SiOPR3* plants showed an expression of this gene three times higher than wild type plants. (Figure 3A). As expected, *SiOPR3* expression that was detected in wild-type plants was strongly reduced in the *SiOPR3* plants in the absence of infection. Moreover, inoculation with *Pst* significantly increased the *SiOPR3* expression in WT plants, while *SiOPR3* induction upon infection was severely reduced in the transgenic plants (Figure 3B).

We next studied the expression of two marker genes of the SA pathway. We observed that plants inoculated with *Pst* showed higher levels of *PR1* and *PR5* gene expression (Figure 3C,D). In both cases, the expression of these genes is two-fold higher in the silenced plants when compared to WT plants. In the same way, in non-inoculated plants, the expression of these genes is significantly higher than in wild type plants (Figure 3C,D).

We further extended our analysis to ABA marker gene, *ASR1* (Figure 3E). The results show an increase in the expression of this gene in silenced plants after inoculation. This correlated with the accumulation of ABA that was observed at this time point.

### 2.4. Bacterial Virulence Genes Showed Different Expression Depending on the Plant Host

**Figure 4:**
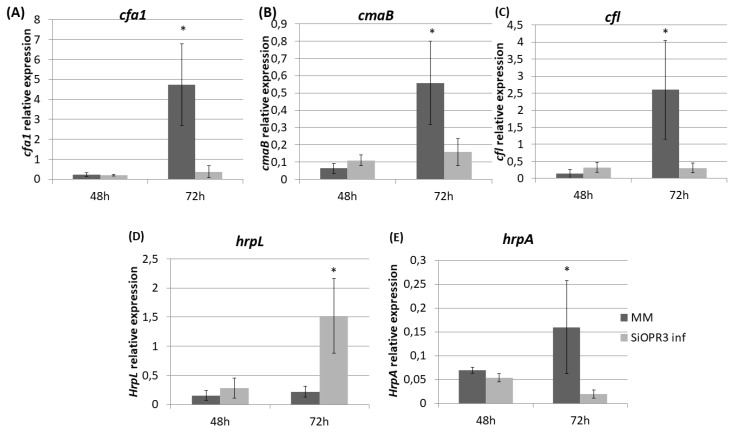
Expression of coronatine (COR) synthesis genes and of type III secretion system-related genes from *Pst* extracted from infected leaves of MM and SiOPR3 plants. The transcript levels of cfa1 (**A**), cmaB (**B**), cfl (**C**), hrpL (**D**), and hrpA (**E**) were examined at 48 and 72 h postinoculation, using qRTPCR with specific primers. The recA gene was used as an endogenous reference gene. The results represent the means and standard error from three different experiments.

We determined the expression of genes related to the pathogenesis and survival of *Pst* DC3000, such as coronatine synthesis-related genes (*cfa1, cmaB* and *cfl*), and genes that were selected as representative of the type III secretion system and the type III secretion system-associated pilus, respectively (*hrpL* and *hrpA*), in order to study the influence of the hormonal balance of the host plant in the virulence of the bacteria. To achieve this goal, the bacteria were extracted from both wild type and *SiOPR3* plants and bacterial genes expression was analyzed at 48 and 72 hpi (Figure 4). No differences were observed in gene expression at 48 hpi. However, the results showed that the transcription levels of *cfa1, cmaB,* and *cfl* were higher in the bacteria from WT plants when compared with those that were extracted from silenced plants at 72 hpi (Figure 4A–C).

On the other hand, we observed a strong induction of *hrpL* in bacteria that were extracted from *SiOPR3* plants in comparison with control plants (Figure 4D). In contrast, the expression of *hrpA*, the major component of the type III secretion system-associated pilus, was stronger in the bacteria from WT plants (Figure 4E).

### 2.5. JA Treatment Restores Susceptibility Phenotype against Pst in SiOPR3 Plants

**Figure 5:**
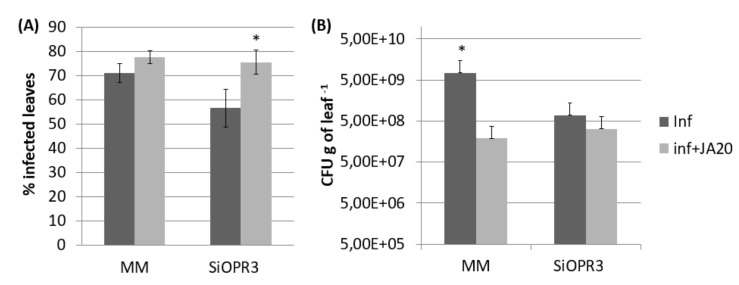
Effect of JA treatment on the response of MM and SiOPR3 transgenic plants to *Pst*. Disease symptoms were measured 72 h after infection in JA-treated and untreated plants as described in Figure 1. The percentage of dark brown spots on the leaf surface (**A**) and the bacterial populations (**B**) are shown. The data show the average values ± standard error (*n* = 20). The asterisks represent statistically significant differences (*p* < 0.05; least-significant difference test).

The results that were observed from gene expression and hormonal levels analyses prompted us to investigate whether exogenous application of JA to *SiOPR3* plants could restore their susceptibility to *Pst*. The application of JA caused a significant increase in the susceptibility to this pathogen in both wild type and *SiOPR3* plants (Figure 5A). However, when we studied the number of CFU g^−1^ of leaf we observed that in both, wild type and silenced plants, the number of bacteria is reduced in comparison with plants that were non-treated with JA (Figure 5B). Altogether, these results indicate that the presence of JA allows the bacteria to produce more damage with less population.

Moreover, in vitro tests were carried out to ascertain whether JA presence had a negative effect on the bacteria due to the decrease in the bacterial population observed in the plants treated with JA. For this purpose, the growth of the bacteria was monitored in LB medium that was amended with the same concentration of JA used for the treatments in planta as well as two concentrations below. The results showed that none of the tested concentrations had a negative effect on the bacteria. Therefore, the hypothesis that the decrease that was observed in bacterial growth in the plants treated with JA is due to the presence of JA was dismissed (data not shown).

### 2.6. The Expression of Bacterial Genes is Altered by JA Treatment

The results that were observed after JA treatment prompted us to examine the expression of the genes analyzed above after the infection of wild type and *SiOPR3* plants with *Pst* to determine whether their expression was altered by the treatment.

**Figure 6:**
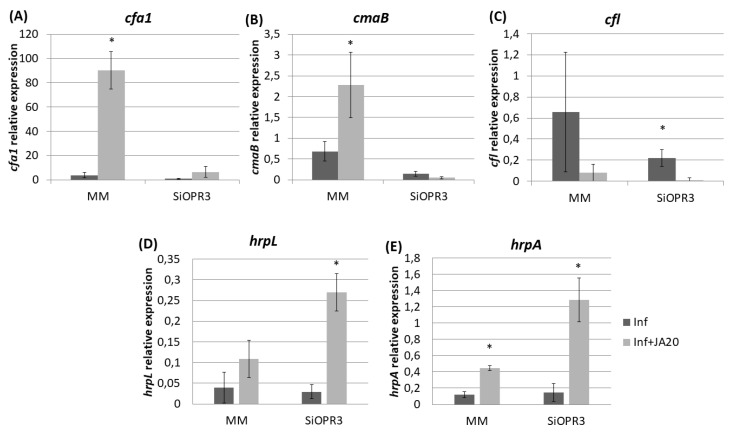
Relative expression of COR synthesis genes and of marker genes of the type III secretion system and the type III secretion system-associated pilus in response to JA treatment. Template cDNAs were generated from the total RNA extracted from bacteria recovered from JA-treated and untreated MM and SiOPR3 plants. The relative expression levels of *cfa1* (**A**), *cmaB* (**B**), *cfl* (**C**), *hrpL* (**D**), and *hrpA* (**E**) were determined and normalized to those of *recA*. The results represent the means and standard error from three different experiments.

The transcription levels of *cfa1* were increased in the bacteria that were extracted from both WT and *SiOPR3* plants after JA treatment (Figure 6A) However, *cfa1* levels were higher in the bacteria extracted from WT plants compared with those extracted from *SiOPR3* plants upon treatment. A similar behavior was observed for *cmaB* gene in the bacteria that were extracted from wild type plants after JA treatment (Figure 6B). *cmaB* expression was lower in the bacteria extracted from treated *SiOPR3* plants, on the contrary to what observed for *cfa1* gene. The values of *cfl* were not affected by the JA treatment (Figure 6C).

On the other hand, when we focused on the genes selected as representative of the type III secretion system and the type III secretion system-associated pilus, we observed that the treatment with JA also enhances the expression of these genes, enhancement, which is much higher in *SiOPR3* plants than in wild type plants (Figure 6D,E).

## 3. Discussion

Jasmonic acid is one of the main phytohormones that orchestrate the resistance against biotic stresses. Despite that, usually, the resistance that is mediated by JA is related to necrotrophic pathogens and herbivores [25], this pathway also plays an important role in the susceptibility against *Pst*. During the process of invasion, *Pst* releases Coronatine, a phytotoxin that mimics the structure of JA-Ile and is thus able to bind JA-receptor COR-insensitive 1 (COI1), activating the JA mediated responses. The presence of JA-Ile or COR activates the expression of the transcription factor JASMONIC ACID2-LIKE, which binds to and activates the expression of SAMT1 and SAMT2, which induces the deactivation of SA by methylation, producing the inhibition of SA responses and promoting stomatal opening [26]. In the same way, it has been described that, in Arabidopsis, the activation of the JA pathway mediated by the application of E-2-hexenal promotes susceptibility to *Pst* [27]. On the other hand, the OPDA, which is a precursor of JA, also plays an important role in plant defense [8]. In Arabidopsis, the characterization of *OPR3* mutants showed that OPDA upregulates COI1-dependent genes, as well as COI1-independent genes that do not respond to JA [8,9].

Previous results showed that *SiOPR3* mutants showed enhanced susceptibility against *Botrytis cinerea* accompanied by a reduction of OPDA and JA-Ile levels [16]. Interestingly, OPDA, but not JA, treatment restored basal resistance to *B. cinerea* and induced callose deposition in SiOPR3 transgenic plants, confirming, this way, the importance of OPDA in the defense of tomato plants against this necrotrophic pathogen. However, as expected, when we analyzed the behavior of the *SiOPR3* transgenic plants that were inoculated with a biotrophic pathogen, we observed different results from those observed against necrotrophic pathogens. In this case, our results showed that the *SiOPR3* transgenic plants are more resistant to *Pst* when compared to WT plants. This resistance was accompanied by an enhancement of OPDA levels, as well as reduced levels of JA and JA-Ile.

In this way, the obtained results could be due either to a positive effect of OPDA by itself or to a reduction of JA and JA derived metabolites, which would impair the mechanism by which the SA responses are blocked, triggering an enhanced defense against biotrophic pathogens. Interestingly, an increase of JA-Ile was observed in *SiOPR3* plants. An alternative pathway of JA synthesis was recently described by Chini et al. [28], which could explain the increase of JA-Ile in silenced plants, despite that the silencing of *OPR3* gene was previously demonstrated on these plants.

It is well described that *Pst* releases COR and other effectors, such as HopZ1a promoting the JA derived responses, in order to block the SA pathways [29,30]. The main action of COR is to bind with SFC^COI1^ receptor complex. This binding triggers the degradation of JAZ, releasing TFs, such as *MYC2*, *bHLH*, and *MYBs*, activating, by this mechanism, the expression of JA-responsive genes [31]. Moreover, it also has been observed in Arabidopsis that the release of COR is able to repress the SA responses, even in JA mutants. Our results showed that *SiOPR3* display higher levels of SA responsive genes *PR1* and *PR5*, which suggests that the mechanism to activate the JA responses downstream COI1 could be compromised.

We analyzed the expression of the bacterial genes that are involved in its virulence system, like *cfa1*, *cmaB*, and *cfl,* which are responsible for COR biosynthesis, in order to clarify this point. We observed that the bacteria that were recovered from *SiOPR3* plants showed much lower expression of coronatine synthesis genes compared with bacteria recovered from wild type plants. This could explain the lower infection observed in silenced plants, since coronatine plays an important role in the establishment of the disease.

Previous studies have demonstrated that the expression of COR biosynthesis genes is induced when the bacterium is in contact with the leaf, leaf crude extract, or apoplastic fluid [32,33]. Consequently, it was suggested that the perception of a complex system involving several organic compounds common in tomato plants regulates the bacterial activation of the synthesis of COR [32]. According to this, the reduction of the expression of COR biosynthesis observed in *SiOPR3* plants suggests that JA, JA-Ile or any metabolite downstream of *OPR3* gene could be part of this complex signaling to activate the COR.

On the contrary, the expression of the *hrpL* gene, which is a transcription sigma factor that activates the expression of the Hrp/Hrc type III secretion system, and several effector proteins are higher in silenced plants. The fact that the expression of this gene is not correlated with the other virulence factors analyzed has been observed in previous studies using hexanoic acid to prevent the bacterial growth [34]. This suggests that HrpL could regulate other responses to stress, to ensure the survival of the bacteria.

The exogenous application of JA results in a restoration of susceptibility phenotype. This result is not surprising, since, as stated above, it has been widely described that the jasmonic acid is mainly involved in plant defense against insects and necrotrophic pathogens, and its presence generally promotes *Pst* infection through the antagonistic relationship between SA and JA signaling [35]. Interestingly, the increase that was observed in the symptoms was not accompanied by a growth of the bacterial population neither in WT plants nor in the silenced plants. We focused on the gene expression of *Pst* from wild type and silenced plants in order to explain this reduction in the bacterial growth. We observed that the exogenous application of JA induces the expression of *Cfa* and *cmaB*, genes, which could explain the enhancement of susceptibility through a higher COR production. However, in *SiOPR3* treated plants, the expression of the c*fa1* and *cmaB* genes is much lower than in WT plants.

The mechanism behind the induction of COR genes by the application of JA has not been previously described. As stated above, it has been described that the activation of the synthesis of COR is regulated by the perception of a complex system that involves several organic compounds [32,36]. In this way, our results may suggest that the perception of JA could play a role as a COR inducer. Moreover, the fact that the exogenous treatment of JA induces c*fa1* but not *cmaB* in bacteria that were recovered from *SiOPR3* plants suggest that the perception either requires a complete JA biosynthetic pathway or is mediated by a metabolite downstream *OPR3*.

Concerning biological regulation, the activation of Cfa synthesis upon recognition of JA or JA derived molecules could be related with an attempt to control the expression of determined JA responsive genes. In this way, it has been previously described that different conjugates of Cfa with different amino acids, such as Cfa-Ala, Cfa-Val, Cfa-Leu, or Cfa-Ile, showed differences in the regulation of JA-responsive genes [37]. In Arabidopsis, Cfa-Leu was able to induce early and late JA-responsive genes, whereas Cfa-Met exhibited stronger activity activating JA-responsive genes, especially *AOS*. Besides, Cfa-Ala and Cfa-Val showed higher activity inducing JA-responsive genes, such as *PDF1.2* and *LOX2* [38]. Hence, by the release of COR, or its moieties Cfa and Cma, the bacteria not only pretend to activate JA derived responses, but also pretend to perform a fine-tuning of JA derived responses in its favor.

On the other hand, when we analyzed the genes that are involved in other virulence mechanisms, such as *hrpL* and *hrpA*, we observed that their expression is increased in plants that are treated with JA and this increase was higher in *SiOPR3* plants. The importance of type III secretion system and the effectors in the establishment of infection was previously demonstrated [23,24]. Moreover, Zhao et al. suggested the relation of the JA pathways and type III secretion system [39]. In this work, they showed that the type III secretion system appears to influence the induction of some JA/wound response genes, but not the contrary, as observed in the present study.

The lack of differences in bacterial populations between the treated plants suggests that the different induced virulence mechanisms, coronatine, and the type III secretion system, compensate each other and the disease could develop with the same intensity in WT and silenced plants respectively. Dudnik and Dudler [40] showed that the type III secretion system is the key pathogenicity determinant in *Pseudomonas.* Thus, the considerable increase in the genes that are involved in this system could cause an increase in virulence or the inhibition of plant defense responses [24], and even though the bacteria are present in a lower number, they are more active and, therefore, the level of virulence, and together with it the symptoms, can be elevated.

Taken together, our results show that the *SiOPR3* plants are more resistant to *Pst* than wild type plants, which indicates that the complete JA biosynthetic pathway plays an important role in the virulence of *Pst* in tomato plants. Interestingly, we observed that COR biosynthetic genes showed higher expression in wild type plants than in *SiOPR3,* which suggests that JA or JA derived metabolites might be necessary for the induction of COR. The exogenous application of JA was able to restore the susceptibility phenotype in silenced plants. However, the expression of COR synthesis genes in *SiOPR3* were not restored after treatment, which reinforced the hypothesis that JA or JA derived metabolite might play a role in the induction of COR. However, the application of exogenous JA was sufficient for inducing the expression of Type III secretion system genes. In conclusion, the virulence of *Pst* is dependent of a complete JA biosynthetic pathway, not only due the well-known antagonism between JA and SA responses, but also due a possible effect in the regulation of bacterial gene expression.

## 4. Materials and Methods

### 4.1. Plant Material and Bacterial Strains

The tomato seeds (*Solanum lycopersicum* Mill. cv. Money Maker) and seeds of silenced plants *SiOPR3* were surface-sterilized and germinated in vitro, in culture plates containing solid MS medium with Gamborg vitamins and supplemented with 10 g L^−1^ sucrose and with kanamycin (50 mg L^−1^), as is described in Scaslchi et al. [16]. 15-day-old plants were transferred to pots with sterilized vermiculite and maintained in a growth chamber under the following environmental conditions: light/dark cycle of 16/8 h, temperature of 24/18 °C, light intensity of 200 μmol m^−2^s ^−1^, and 60% relative humidity. The plants were irrigated with Hoagland solution [41]. The pH of the nutrient solution was adjusted to 5.8–6.0 with 1 mM KOH.

The *Pst* strain DC3000, rifampicin resistant was used in this study [42]. The bacterial strain was grown in King B medium (KB) with rifampicin being added (50 mg mL^−1^; Sigma Aldrich, San Luis, MO, USA) at 28 °C in darkness for 24 h.

### 4.2. Plant Inoculation and Determination of Disease Severity and Disease Incidence

Four-week-old tomato plants of *Solanum lycopersicum* Mill. cv. Money Maker and *SiOPR3* plants were inoculated, as described below. The bacterial suspension used for leaves inoculation was obtained from a 24 h culture, by resuspending the bacteria in sterile MgSO_4_ (10 mM) solution containing 0.01% of the surfactant Silwet L-77 (Osi Specialties, Danbury, CT, USA) and adjusted to 5 × 10^5^ colony-forming units (CFU) mL^−1^ while using a spectophotometer. The inoculation was performed by dipping the third and fourth leaf of each plant in the bacterial suspension during 3 s.

The third and fourth leaves that were inoculated of each plant were selected at 72 h post inoculation (hpi) and evaluated by visual observation to determine the percentage of dark brown spots on the leaf surface to determine disease severity (DS). Disease incidence (DI) was determined by counting the number of CFU of *Pst* strain DC3000 per gram of leaves determined in KB medium by the technique of serial dilutions, as described in Scalschi et al. [34]. The number of CFU g^−1^ was counted after 24 h of incubation at 28 °C in the dark. More than three experiments were performed under the described conditions.

### 4.3. Quantitative RT-PCR

The leaves were ground in liquid nitrogen and RNA was extracted while using E:Z:N:A Plant RNA kit OMEGA biotek (http://www.omegabiotek.com), according to the manufacturer’s instructions. 1 μg of total RNA after DNase treatment (Promega, http://www.promega.com) was reverse transcribed into cDNA while using an oligodT primer and primescript RT enzyme mix (Primescript RT reagent kit, TaKaRa Bio Europe, Saint-Germain-en-Laye, France), as described by the manufacturer.

For the analysis of the bacterial response, the bacteria were recovered from the infected leaves and RNA was extracted, as described by Yu et al. [43]. Reverse transcription was carried out from 1 µg of total RNA for gene expression analysis, by using primescript RT enzyme mix and random hexamer primers (Primescript RT reagent kit, TaKaRa Bio Europe, Saint-Germain-en-Laye, France). Quantitative real-time PCR was performed with Maxima SYBR Green/ROX qPCR Master Mix (Thermo Fisher, Waltham, MA, USA) in a StepOneTM Real-Time PCR System (Thermo Fisher, Waltham, MA, USA). The forward and reverse primers (10 μM) were added to 5 μL of Maxima SYBR Green/ROX qPCR Master Mix (Thermo Fisher, Waltham, MA, USA), as well as 1 μL of diluted cDNA and Milli-Q sterile water up to a total reaction volume of 10 μL.

For the analysis of plant genes, primers that were designed for *AOC*, *OPR3*, *PR1*, *PR5*, and *ASR1*, were used. Actin was used as a control to normalize the gene expression in each sample. Flors et al. described the primers used for actin and *PR1* [44], for *AOC* and *OPR3* by Uppalapati et al. [45], and for *PR1* and *PR5* by Scalschi et al. [34]. For bacterial gene expression analysis, specific primers for the following genes were used: *cfa1*, *cmaB,* and *cfl* (implied in Coronatine synthesis) and *hrpL* and *hrpA* (control and synthesis of the Type III secretion system). Scalschi et al. described these primers [46]. The relative levels of the monitored genes were normalized with *recA,* which was used as an internal reference.

### 4.4. Hormone Analysis

Fresh material was frozen in liquid nitrogen, ground, and freeze-dried. Dry leaf tissue (0.05 g) was homogenized in 1.5 mL of ultrapure water, and a mixture of internal standards was added before the extraction: d6-ABA, d4-SA, dihydrojasmonic acid, and propylparaben. The samples were centrifuged at 5000 rpm for 45 min. at 4 °C. The supernatant was partitioned against diethylether, dried in a speed vacuum, and then resuspended in 90:10 H_2_O:MeOH. After extraction, a 20 μL aliquot was directly injected into an Acquity ultra-performance liquid chromatography system (UPLC) with an ACQUITY UPLC BEH C18 column (1.7 μm 2.1 × 50 mm) (Waters, Mildford, MA, USA), which was interfaced with a triple quadrupole mass spectrometer (TQD, Waters, Manchester, UK). MASSLYNX NT software version 4.1 (Micromass Waters, Milford, MA, USA) was used to process the quantitative data from the calibration standards and plant samples.

### 4.5. In vitro Bacterial Growth Assay

For the analysis of the direct effect of OPDA and JA on the bacteria in vitro, *Pst* were grown in LB for control and LB with three different concentrations of OPDA (0.1 µM, 1 µM, and 5 µM) or with three different concentrations of JA (5 µM, 10 µM, and 20 µM) during 24 h at 28 °C in the dark. The growth assay was carried in a microtiter plate, in a total volume of 200 μL LB with or without OPDA or JA, while using an initial bacterial concentration of about 1 × 10^6^ CFU mL^−1^. Bacterial growth was monitored by measuring the optical density in a microplate reader (MB-580, Heales) at 24 hpi in the medium. Eight independent replicates were performed for each condition.

### 4.6. Chemical Treatments in Plants

For chemical treatments, the plants were grown and inoculated, as described for Section 4.2. Plant inoculation and determination of disease severity and disease incidence.

Two days before inoculation, the third and fourth leaf of each plant plants were spray-treated with 20 μm JA. JA was purchased from Sigma-Aldrich (San Luis, MO, USA). The plants were harvested and assessed for disease symptoms 72 h after inoculation. For the analysis of the bacterial response, the bacteria were recovered from the infected leaves, both treated and untreated, and RNA extraction and qPCR were performed, as described above.

### 4.7. Statistical Analysis

All experiments were conducted at least three times. All data were tested for normality and homogeneity of variances. When ANOVA showed significant differences between variables, mean values were compared using the Fisher’s least significant difference (LSD) test at the 95% confidence. Statistical analyses were performed using the software Statgraphics Centurion XVI (Statpoint Technologies, Warrenton, VA, USA).
